# Cure of Opium Habit

**Published:** 1879-02

**Authors:** Wm. O’Daniel

**Affiliations:** Bullards, Ga.


### CURE OF OPIUM HABIT.

By Wm. O'DANIEL, M.D., Bullards, Ga.

In the January number of your Journal, I see that you are requested by an M.D. to give the treatment of the opium habit, or the antidote to the same. As there is much written upon the subject these days, and as there seems to be quite a diversity of opinion as to the most successful plan of treatment, I will give the treatment of a single case, which may be considered of some importance.

I think, as a rule, the reduction system is not a successful one, not because it is not a good one, but because patients will not conscientiously and strictly adhere to and submit to it. It is, in my opinion, the most rational plan and the least hurtful to the patient, if the plan could always be enforced by a physician; but patients and nurses rarely ever follow such directions honestly. Therefore, when it is impossible for the attending physician to have his directions properly executed, I am decidedly in favor of abandoning the habit altogether. True, it is sometimes very painful to the patient. I have known many victims of the opium habit, as well as those of the whisky habit, to try unsuccessfully the reduction plan, or "tapering off" plan, as they style it, and I have frequently known the tapering end to become the larger, simply for the want of positive determination to control when the system absolutely calls for a stimulant.

Mr. P., of Twiggs county, an athletic man of about forty years, weighing two hundred pounds, in 1869 commenced to relieve every ill and unpleasant sensation by the use of morphine. He soon found himself a confirmed victim of the destructive habit—so much so that he was unable to keep awake whenever still, and his neighbors and family began to discover mental aberrations.

The habit continued gradually to grow until less than two or three drams per week were totally insufficient to produce the desired effect—he always taking it per orem. Of course he saw that the cultivation of the habit promised nothing but ruin. In the summer of 1877, after having been a slave to the habit for eight years, he determined to abandon it, even at the risk of his life. He soon found himself confined to his bed, and sent for me, and told me that he had resolved to take no more morphine, and that he desired to place himself under my professional care. I told him that I had no confidence in his fixedness of purpose and will to leave off entirely the habit; but if he was in earnest, I would endeavor to aid him in the good work which he had resolved to perform. Upon examination I found him with a distressing diarrhœa, profuse perspiration, exceedingly nervous, and frequently he could not concentrate his thoughts sufficiently long to complete a sentence, *with general prostration.* I put him upon alcoholic stimulants, chloroform, chloral hydrate, bromide of potassum, quinine, strychnine, capsicum, etc., during his sickness (for he was severely sick), of course alternating and using those which I thought had the most happy effect. But he apparently grew worse for two weeks, was very much reduced in flesh, had no appetite, and several times I debated in my mind the propriety of returning him to the habit, and leaving him to work out his own destiny. Until he commenced to convalesce he weighed less than one hundred pounds, not half what he weighed when he commenced the habit; but finally he began to improve, and in six weeks he was cured of the opium habit, and had no desire for any kind of a stimulant, and is to-day as fine a specimen of health as any country ever produced. I do think the case in question was, during his attempt to abandon the habit of opium-taking, the greatest sufferer I ever knew. He complained of the most excruciating pains in his limbs, and indeed in his whole body. He said they were equal in every way to the worst form of toothache, which is said by a poet of reputation to be "the hell of all diseases."

